# Double-stranded DNA virioplankton dynamics and reproductive strategies in the oligotrophic open ocean water column

**DOI:** 10.1038/s41396-020-0604-8

**Published:** 2020-02-14

**Authors:** Elaine Luo, John M. Eppley, Anna E. Romano, Daniel R. Mende, Edward F. DeLong

**Affiliations:** Daniel K. Inouye Center for Microbial Oceanography: Research and Education (C-MORE), University of Hawaii, Honolulu, HI 96822 USA

**Keywords:** Metagenomics, Microbial ecology

## Abstract

Microbial communities are critical to ecosystem dynamics and biogeochemical cycling in the open oceans. Viruses are essential elements of these communities, influencing the productivity, diversity, and evolution of cellular hosts. To further explore the natural history and ecology of open-ocean viruses, we surveyed the spatiotemporal dynamics of double-stranded DNA (dsDNA) viruses in both virioplankton and bacterioplankton size fractions in the North Pacific Subtropical Gyre, one of the largest biomes on the planet. Assembly and clustering of viral genomes revealed a peak in virioplankton diversity at the base of the euphotic zone, where virus populations and host species richness both reached their maxima. Simultaneous characterization of both extracellular and intracellular viruses suggested depth-specific reproductive strategies. In particular, analyses indicated elevated lytic interactions in the mixed layer, more temporally variable temperate phage interactions at the base of the euphotic zone, and increased lysogeny in the mesopelagic ocean. Furthermore, the depth variability of auxiliary metabolic genes suggested habitat-specific strategies for viral influence on light-energy, nitrogen, and phosphorus acquisition during host infection. Most virus populations were temporally persistent over several years in this environment at the 95% nucleic acid identity level. In total, our analyses revealed variable distributional patterns and diverse reproductive and metabolic strategies of virus populations in the open-ocean water column.

## Introduction

Viruses represent dynamic reservoirs of unexplored genetic diversity. On average an order of magnitude more abundant than cellular organisms [1], viruses occur at ~10^7^/mL in the surface layer of open oceans covering ~40% of Earth [2]. In this environment, dsDNA bacteriophages infect key microbial groups, including oxygenic photoautotrophs such as *Prochlorococcus* and *Synechococcus* (e.g., [3, 4]) and common bacterial heterotrophs such as *Pelagibacter* (SAR11), *Puniceispirillum* (SAR116), *Roseobacter*, and *Alteromonas* (e.g., [5–8]). Viruses can lyse their hosts at estimated rates of 20–40% per day [9, 10], potentially contributing as much as 145 gigatonnes to annual global carbon flux [11, 12]. Viruses also influence the diversity and biogeochemistry of marine ecosystems by carrying auxiliary metabolic genes (AMGs) that manipulate host metabolism during infection (reviewed in [13]).

Recent developments in high-throughput DNA sequencing have allowed for exploration of viral diversity at unprecedented scales. The continuing description of viral genetic diversity highlights the importance of further cultivation-independent in situ characterization of environmental viral populations. Metagenomic virus surveys have focused on characterizing geographic variability across surface oceans [14–17], temporal variability at the surface ocean [18–21], and vertical variability across depth profiles [22–24]. Coupled studies of viral and host dynamics in both space and time at well-defined sample sites [25–27] further have the potential to provide additional perspective on the dynamics, patterns and consequences of viral diversity.

To further explore viral diversity, environmental distributions, and dynamics in the open ocean, we characterized virus genomes from seawater in virus-enriched (0.02–0.2 μm) and cell-enriched (>0.2 μm) size fractions over time and depth in the North Pacific Subtropical Gyre (NPSG). The approach facilitated the exploration of both extracellular virioplankton particles as well as cell-associated phages, to better characterize reproductive and metabolic strategies of viruses in the open ocean. We use the term “reproductive strategies” here to refer life history differences between strictly lytic viruses with a singular strategy of host lysis, versus temperate phages that in addition to the lytic cycle, have the potential to either integrate into their host’s genome or reside as an extrachromosomal element. In this study, we analyzed samples collected over 1.5 years over 12 depths (5–500 m) to characterize dsDNA viruses in both virioplankton and bacterioplankton size fractions. This virus genome dataset (referred to here as the ALOHA 2.0 viral database) provides new perspectives on the diversity, reproductive strategies, gene content, and ecology of viruses in the open ocean.

## Methods

A schematic overview of our workflow is presented in Fig. S1.

### Sample collection, extraction, and sequencing

Station ALOHA (22°45′ N, 158° W), a relatively seasonally stable environment located in the NPSG, is a well-characterized sampling site of the Hawaii Ocean Time-series (HOT) program. The ALOHA 2.0 dataset contains 374 metagenomic samples collected at 12 depths (5, 24, 45, 75, 100, 125, 150, 175, 200, 225, 250, and 500 m) at approximately monthly intervals of 16 timepoints spanning 1.5 years from 2014 to 2016 (Fig. S2). These collections correspond to HOT cruise numbers 267–283, for which physiochemical data are available in Table S1 and on Hawaii Ocean Time-Series HOT-DOGS application (http://hahana.soest.hawaii.edu/hot/hot-dogs).

All samples were collected using the following procedure. 2–4 L of seawater (2 L from 5 to 175 m, 4 L from 200–500 m) were collected using CTD-attached Niskin bottles and filtered, using peristaltic pumps at a flow rate of about 6 L/h, onto a 0.2 μm 25 mm Supor filter (VWR 28147-956) housed in polypropylene filter holder (Cole-Palmer EW-06623-32). These >0.2 μm cell-enriched samples were removed from filter holder and stored in 300 μL of RNALater (Ambion AM7021, Waltham MA) at −80 °C. 1–2 L (1 L from 5–175 m, 2 L from 200–500 m) of the < 0.2 μm filtrate were collected and filtered, using peristaltic pumps at a flow rate of about 1 L/h, onto a 0.02 μm Whatman Anotop filter (VWR 28138-017, Radnor PA). These corresponding 0.2–0.02 μm virus-enriched samples were stored sealed in filter housing at −80 °C. DNA extraction, sequencing, and read quality-control are described in the Supplementary methods.

Quality-controlled reads, on average 9–10 million per sample (Table S1), were assembled within each sample using option “-k 21,33,55,77,99,127” on metaSPAdes v3.10.1 [28], generating a total of 83 million contigs amongst 416 metagenomes (Table S1). All sequences were used for viral reassembly and database curation. For downstream analyses, smaller metagenomes from samples with duplicate sequencing runs and samples with <1 million reads were removed, resulting in a total of 374 metagenomes. Sequences were submitted to NCBI SRA under project number PRJNA352737 and assemblies can be found under BioSample SAMN12604809.

### Viral-specific reassembly

All >3 kb contigs were filtered using VIRSorter v1.03 [29] using the virome database, under regular mode for cell-enriched samples and decontamination mode for virus-enriched samples. Contigs from all identified viral categories were retained (Table S2). BWA-MEM v0.7.15 (Li 2013) and msamtools [30] was used to identify 809 million reads mapping to these putative viral contigs at >95% average nucleotide identity (ANI) across >45 bp (Table S2). Viral reads were reassembled using metaSPAdes v3.11.1, which was chosen due to improved genome recovery and low rate of generating false apparent circularity [31]. Reads from contigs with >10 coverage, a threshold representing 99% genome recovery [31], were pooled across each depth for 12 reassemblies. Reads from contigs with <10 coverage were pooled across all samples into a low-coverage reassembly to improve genome recovery.

### ALOHA 2.0 virus database curation

Viral contigs across the 13 reassemblies, along with viral contigs from two previous smaller datasets near Station ALOHA [26, 32], were clustered with cd-hit-est v4.6 [33] at >95% ANI to form 1.5 million nonredundant viral populations. Populations were filtered through VIRSorter and 262 197 putative viruses from all categories were retained. Proteins were predicted using Prodigal v2.6.3 [34] and functionally annotated using HMMer v.3.2 [35] against the PFAM-A v30 database [36]. Populations containing one or more known viral marker proteins were retained (bit score >30 to capsid, head, neck, tail, spike, portal, terminase, clamp loader, T4 proteins, T7 proteins, Mu proteins, excisionase, phage integrase, repressor protein CI, or Cro), resulting in 56,559 high-confidence virus populations ranging from 0.5 to 366 kbp in length. To focus on full genomes or large genomic fragments, we retained only >10 kbp contigs, resulting in 17,369 populations that form the ALOHA 2.0 virus database. Functional annotations were inspected to ensure that no ribosomal proteins are present, with the exception of S21 found also in a cultivated *Pelagibacter* phage, S33 found enriched in aquatic viruses, and L7/12 found in assembled viral contigs [37]. One population containing L11 was retained due to the protein’s proximity and interaction with L7/12 [38].

The high proportion of novel viral diversity in our samples precludes using reference genomes for the detection of chimeras, which are expected to occur at a frequency of ~0.5% using metaSPAdes [31]. As a result, we inspected clusters for chimeric signature through self-alignment using LAST v756 [39] to identify stretches of repeats at >95%ANI across >10 kbp. Fifteen populations displayed this signature and were noted as chimeras (Table S3).

### Genomic completion

We used terminal repeats (apparent circularity) to identify a nonredundant set of complete genomes [40], using a combination of four different methods: i. 439 were identified using Virsorter ii. 411 from check_circularity.pl [41] iii. 790 from LAST to identify overlaps at 95% ANI across 100 bp–10 kbp within 200 bp of both ends; and iv. 1131 from NUCmer v3.1 [42] to identify direct terminal repeats 20bp–10kbp in length within 200 bp of both ends [43]. 1543 complete genomes were identified pooled amongst these four methods (Table S3). LAST was used to assess redundant circularly permuted contigs at 95% ANI across 150 bp, yielding 961 nonredundant complete genomes (16,787 total, Fig. S1).

### Viral and prokaryotic contribution to total DNA

Viral contribution was calculated for each sample as the proportion of reads mapping to the ALOHA 2.0 viral database (>95% across >45 bp). Prokaryotic contribution was calculated for each sample as the proportion of reads classified as bacterial or archaeal with Kaiju v1.6.2 [44].

### Spatiotemporal distribution and abundance

Reads from each sample were mapped using BWA-MEM to virus populations and filtered using msamtools at >95% across >45 bp. Anvi’o v3 [45] was used to calculate coverage profiles for every sample, using interquartile range (IQR) coverage, which diminishes the effect of conserved or hypervariable regions in respectively over- and under-estimating coverage. For analyses including all virus populations, relative nucleotides mapped (nucleotides mapped to population divided by nucleotides mapped across all populations) was used to calculate relative abundances (Tables S4, S5). For analyses including only complete viral genomes, relative coverage (genome coverage divided by total coverage summed across all complete genomes) was used to approximate relative abundances (Tables S6, S7).

To examine temporal persistence, reads from a previous 2010–2011 dataset from Station ALOHA [46] were mapped to the 2014-6 ALOHA 2.0 virus database using BWA-MEM and filtered using msamtools at >95% across >45 bp. Populations with non-zero IQR coverage were considered present in the 2010–2011 dataset (Table S3).

### Characterizing cellular assemblages

COG0012, a universal single-copy marker protein, was used to generate 2568 mOTUs representing cells at the near-species level, at higher resolution than with rRNA-based OTUs [46–48]. One *Crocosphaera* mOTU not previously included was curated, and relative abundances were calculated using relative coverages of reads mapping to these 2569 mOTUs (Table S8).

### Viral population identification

Taxonomy was assigned using protein LAST alignments to known phages in RefSeq84 ([49]; Table S9), as well as five viral metagenomic databases available as of 2018: uvMED [50], uvDEEP [23], GOV [15], EV [16], and MED2017 [24]. To avoid inflating the number of novel populations, we performed broad taxonomic assignments at >60% average amino acid identity (AAI) across >50% of proteins to any reference genome or contig, with the best hit assigned based on the highest AAI (Table S3). A broader cut-off of >50% of proteins at any AAI was used to identify phages infecting heterotrophic bacterioplankton in RefSeq84, due to lower sequence representation than picocyanophages in these databases (Table S3).

Proteins were annotated using HMMsearch against PFAM (bit score >30). Proteins with functional domains not found in previously reported datasets [15, 26] were considered novel viral genes (Table S10).

Putative temperate phages were identified using i. functional annotations to identify phages with the genomic potential for lysogeny and ii. VIRSorter to identify integrated prophages. i. Functional annotations identified 922 populations with temperate phage markers (>30 bit score to integrase, excisionase, Cro, or CI repressor). ii. VIRSorter identified prophages from original assemblies, of which 413 temperate phage populations shared significant homology (a minimum of 150 bp at 95% ANI); VIRSorter identified 73 final viral populations as prophages.

### Archaeal virus identification

Putative archaeal viruses were identified in unannotated populations using archaeal protein markers and sequence similarity to Archaea or archaeal viruses in RefSeq84 using archaeal markers (PFAM bit score >30). Populations carrying protein markers included one with AmoC [51], 16 with archaeal holiday junction resolvase, and 37 with MCM DNA helicase of archaeal origin (Table S9), yielding a total of 53 archaeal virus marker populations. 632 populations were identified having ≥1 proteins with top hits (protein–protein LAST) to Archaea or archaeal viruses in the RefSeq84 database [49]. We refined this set with the expectation that archaeal viruses should display high ratio of top protein hits to Archaea and archaeal viruses divided by top hits to Bacteria (AB ratio) and large proportion of proteins without RefSeq84 hits, given the scarcity of open ocean archaeal viruses in current databases. The 53 archaeal virus marker populations all displayed >0.06 AB ratio and >0.8 proportion of proteins without RefSeq84 hits (Fig. S3b). We used modified, conservative cut-offs (>0.5 AB ratio, >0.8 proportion of proteins without RefSeq84 hits) to retain 161 putative archaeal viruses (Fig. S3c). Phylum-level classifications were assigned based on number of protein top hits to Thaumarchaeota and Euryarchaeota. Populations with ties in number of top hits to these phyla were considered unclassified archaeal viruses.

### Eukaryotic virus identification

Putative eukaryotic viruses were identified using the nucleocytoplasmic large DNA virus capsid marker (PFAM bit score >30) and ≥2 proteins with top hits to eukaryotic viruses in RefSeq84 (Table S9). All taxonomic identifications were cross-referenced to confirm that a population is assigned only to one group.

### *Crocosphaera* putative phage identification

Putative *Crocosphaera* phages were identified through a combination of methods. First, alignments to RefSeq84 revealed one putative *Crocosphaera* phage (Table S3) with 11 of 15 proteins hitting to *Crocrosphaera*, and one hit to another Cyanobacteria (Table S9). For independent confirmation, this virus displayed abundance profiles expected from *Crocosphaera* (i.e., summer bloom in the upper ocean). 170 other potential *Crocosphaera* phages that displayed similar spatiotemporal distributions (Fig. S9) were also included as candidates for independent confirmation. Reads from samples capturing a *Crocosphaera* bloom (and presumably its phages) near Station ALOHA around the same time [52] were mapped (>95% across >45 bp) to these 171 populations to confirm presence. 115 candidates that recruited reads at >0 IQR coverage were retained. We then retained only candidates with higher number of top hits to non-*Prochlorococcus*/*Synechococcus* cyanobacterial proteins than top hits to *Prochlorococcus* or *Synechococcus* proteins, for a final set of one “putative” and six “potential” *Crocosphaera* phages (Table S3).

### Viral and prokaryotic diversity

To examine within-sample α-diversity, we calculated the Shannon diversity, evenness, and richness for each sample using the vegan package in R [53]. Respective assemblages were assessed using relative coverages of 16,787 viral populations and 2568 cellular mOTUs.

### VC ratio

We define the Virus:Cell ratio (VC ratio) here as the log-ratio of a population’s relative abundance (nucleotides mapped) in the virus-enriched versus cell-enriched size fractions. To calculate VC ratios, samtools bedcov [54] was used to calculate nucleotides mapping to each population from all samples, normalized to the average library size of 1.3 million nucleotides per sample. Populations with zero nucleotides mapping in any sample were adjusted to one nucleotide for log-ratio calculation. Temporal variability of VC ratios was calculated for each population using the mean-normalized variance of its VC ratios within each depth.

## Results and discussion

A total of 374 metagenomes were generated from seawater sampled at Station ALOHA across 16 timepoints at 12 depths (5–500 m). These samples were used to prepare DNA from cell-enriched >0.2 μm size fractions (encompassing cellular DNA, giant viruses, active infections, prophages, and absorbed phages), and virus-enriched 0.2–0.02 μm size fractions (capturing ultra-small cells, free virus particles, and ultra-small detritus). After metagenome assembly, curation, and classification (Fig. S1), the 4.2 TB of sequence data yielded 16,787 virus populations, with 8079 novel populations not represented in publicly available virus sequences. A total of 961 assembled populations were identified as complete via evidence of their terminal repeats [55, 56]. 1352 populations were identified as putative temperate phages, due to the presence of flanking cellular sequences or diagnostic temperate phage marker genes. Among novel viruses, 29 putative temperate *Pelagibacter* and *Puniceispirillum* phages were identified, along with 12 complete archaeal virus genomes, 25 genomic fragments of eukaryotic viruses, and a putative *Crocosphaera* phage-like element (Fig. S4, S5). The curated viral populations contained 236 novel viral proteins (Table S10) not previously reported from marine environments [15, 26]. These proteins included auxiliary metabolic genes in nitrogen (nitronate monooxygenase) and phosphorus cycling (transporter), phage holin, a toxin–antitoxin system (Fig. S4), and CRISPR-associated proteins.

### Viral DNA contribution to total DNA

**Fig. 1:**
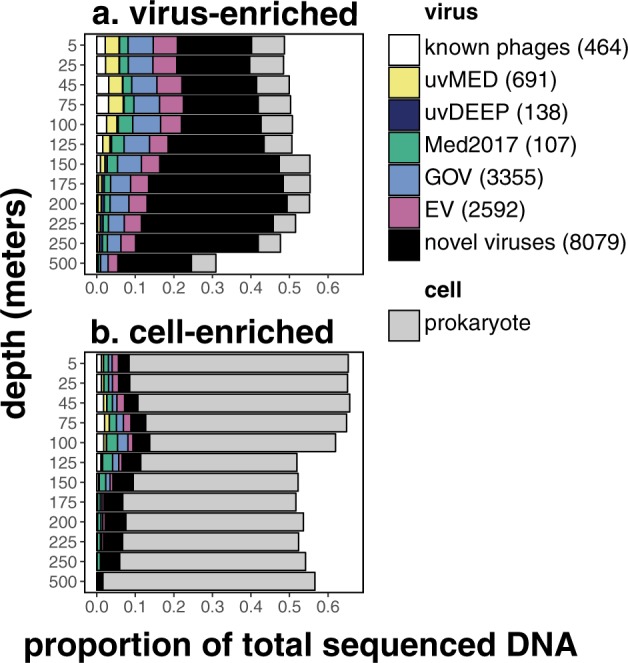
Depth profiles of time-averaged viral and prokaryotic contributions to total sequenced DNA in **a** virus-enriched and **b** cell-enriched size fractions. Relative abundances of viral populations are colored by average amino acid identity (AAI) (>60% AAI across >50% genes) to known phages in RefSeq, and five other viral metagenomic datasets: uvMED [50], uvDEEP [23], Med2017 [24], GOV [15], and EV [16]. Legend shows number of populations identified in each category.

On average across all depths, the ALOHA 2.0 viral dataset recruited 42% of reads from the virus-enriched fraction and 8% of reads from the cell-enriched fraction (Fig. 1). Assuming that viruses account for 56% of the total DNA in the <0.22 μm fraction [57], our dataset recovered on average 75% of the total viral DNA from this habitat. In the virus-enriched fraction, viral contribution total DNA peaked near the base of the euphotic zone, 150–250 m. This increase in relative viral DNA contribution may reflect increased cellular turnover and viral production, increased temperate phage induction (described later), or decreased decay rates due to lower ambient UV light fluxes and temperature at these depths [58]. In the cell-enriched fraction, relative viral contribution to total DNA peaked near the deep chlorophyll maximum (DCM) around 100 m, extending previous observations of subsurface cyanophage maxima in cell-enriched fractions at Station ALOHA [59]. Since other sources of DNA contribution are mostly cellular in origin, the increase in ratio of viral-to-bacterial DNA likely indicates a combination of increased active infections and prophages in these cell populations.

### Viral diversity hotspot at the base of the euphotic zone

**Fig. 2:**
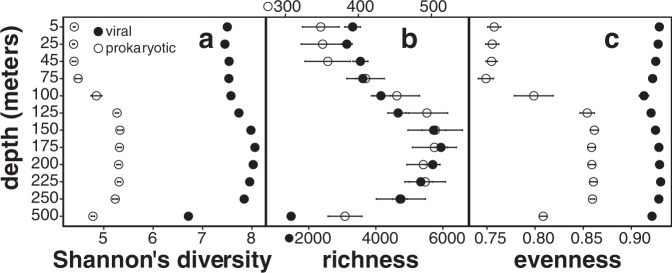
α-diversity depth profiles of viral and prokaryotic assemblages: **a** Shannon diversity, **b** richness (number of populations), and **c** evenness (Shannon diversity divided by log richness). Solid and open circles represent viral and prokaryotic assemblages, respectively, averaged through time (mean ± SE).

**Fig. 3:**
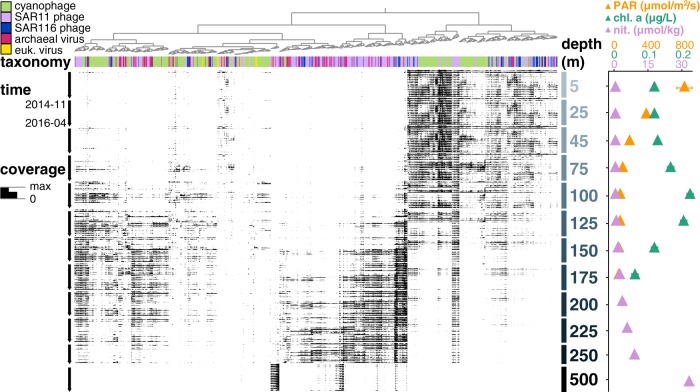
Spatiotemporal distributions of annotated virus populations present in the virus-enriched size fraction. Each node on the top dendrogram and its associated column represents the coverage profile of one virus population, colored by its corresponding taxonomic annotation. Rows represent individual samples that are horizontally ordered by depth and time. The height of the black bar in every sample shows mean interquartile range (IQR) coverage of every population, normalized to the maximum IQR coverage in that sample. Time-averaged depth profiles (mean ± SE) of environmental variables of photosynthetically active radiation (PAR), fluorometric chlorophyll a, and inorganic nitrogen are shown in colored triangles on the right panel (data retrieved from Hawaii Ocean Time-Series HOT-DOGS application).

Within-sample α-diversity and richness revealed hotspots for virioplankton diversity, and confirmed it for prokaryotic diversity [60], at the base of the euphotic zone between 150 and 250 m (Fig. 2). This peak in diversity could reflect both habitat variability and transitions in microbial metabolic diversity. For example, in this environment, photoautotrophic cyanobacteria dominate in the photic zone, while chemolithotrophic ammonium-oxidizing Thaumarchaeota and (presumptive) heterotrophic Euryarchaeota are both more abundant in deep waters. However, both cyanobacterial and archaeal groups do co-exist at transitional depths in and just below the deep chlorophyll maximum [60]. Similarly, while cyanophages were found predominantly in shallow waters and archaeal viruses mostly below the photic zone, both cyanophages and archaeal viruses were found at transitional depths between 150 and 250 m (annotations shown on Fig. 3, top bar). Previously uncharacterized viruses also increased in abundance below the upper surface waters, and represented >50% of viral assemblages below 125 m (Fig. 1). Elevated richness in oligotrophic picoplankton communities just below the photic zone was evident not only in Bacteria and Archaea, but also their viruses.

### Depth-dependent patterns in temperate phage integration and induction

**Fig. 4:**
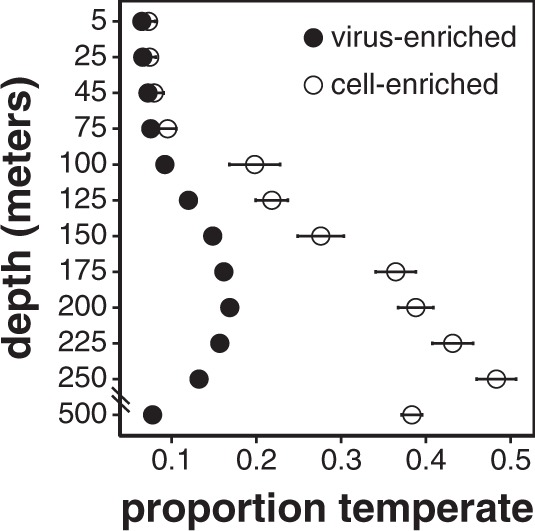
Depth profiles of relative abundances of putative temperate phages. Circles represent time-averaged depth profiles (mean ± SE) of the proportion of all viruses that were identified as temperate in the virus-enriched size fraction (closed circles) and cell-enriched size fraction (open circles).

The community-wide abundances of 1352 putative temperate phages displayed depth-specific patterns (Fig. 4). In cell-enriched samples, we postulate that putative temperate phages represented integrated prophages, which increased at and below the DCM, consistent with previous reports of apparent increased lysogeny in deeper waters [26, 61, 62]. Depth-dependent changes in viral reproductive strategies were also evident within groups at the genus level, particularly in SAR11 phages with broad depth ranges (Fig. S7). Our results suggest that community-wide changes in reproductive strategies are driven not only by specific host groups, but also partly by environmental variability along the water column. Given that cellular host abundance generally decreases with depth (Fig. S2c), our results do not appear to support the piggyback-the-winner [63] hypothesis in this oligotrophic pelagic habitat. It seems probable that virus-host dynamics may have different trajectories in different environmental, biological, and ecological contexts, and may not be driven simply by bulk numerical host-cell and virus-particle ratios alone. In oligotrophic pelagic environments, high prokaryote cell densities in surface waters correspond to smaller average genome sizes [46, 64]. Within and below the DCM, low cell densities correspond to larger average genome sizes [46]. Host cell genome sizes (and therefore genomic “real estate” available for prophage, genomic islands and other mobile elements), rather than cell densities, might drive viral reproductive strategies [65].

**Fig. 5:**
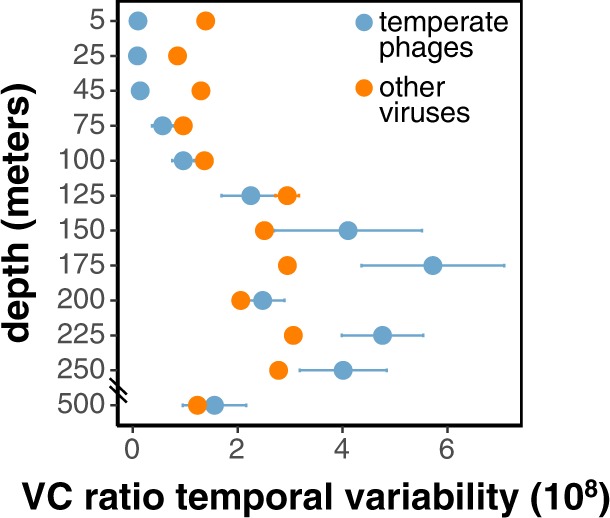
Depth profiles of temporal variabilities of VC ratios (mean ± SE) of 1352 inferred temperate phages (blue) and other viral populations (orange). Higher VC ratio temporal variability indicates episodic production of free viral particles, while lower variability indicates consistent production of free viral particles. Temporal variability of VC ratios is calculated for each population by pooling its VC ratios within each depth to determine the mean-normalized variance.

In virus-enriched samples, we postulate that temperate phage signal represents temperate virus particles, which peaked at 150–250 m (Fig. 4). This peak may reflect increased temperate phage productivity relative to deeper waters associated with more slowly growing hosts [66]. Temperate phage production at these intermediate depths appeared to be episodic, as indicated by temporal variability in populations’ extracellular to intracellular abundance (VC) ratios (Fig. 5). Low VC variability reflects phages with constant or no viral particle production. High VC variability indicates phages with more episodic virus particle production. The VC variability of temperate phages peaked at 150–250 m (Fig. 5), consistent with episodic induction and production. Episodic temperate phage induction may be driven by temporal resource variability or host abundance [67–69], or increased cellular stress due to lower light for photoautotrophs at these depths (Fig. S2f).

Given the low abundance of temperate phages in the upper 75 m (Fig. 4), we infer that lytic phage–host interactions were prevalent in surface waters, potentially reflecting consistently high productivity and host abundance that can favor lytic strategies [66, 70–72]. Temperate phage induction and production appeared to peak at the base of the euphotic zone (Figs. 4, 5), potentially reflecting increased environmental variability at these depths that might favor a flexible reproductive strategy [69, 73]. Evidence for host-integrated prophages increased in mesopelagic waters (Fig. 4), possibly reflecting decreases in host productivity and abundance [72, 73], or an increase in genome size that might better accommodate prophages [46, 65]. Using size fractionation to separate intra- and extracellular viruses, our results revealed depth-specific viral reproductive strategies from the surface to mesopelagic ocean.

### VC ratio to confirm genomic temperate phage identification

The VC ratio of each population represents its extracellular to intracellular abundance ratio. On average, temperate phages, or phages persisting intracellularly for long periods before initiating a lytic cycle, are expected to have lower VC ratios relative to strictly lytic phages. Indeed, temperate phages on average displayed lower VC ratios than that of other coexisting populations (Welsh’s *t* test, *p* = 0.02). This trend provided further support for our temperate phage identifications using genomic markers. Nevertheless, intracellular temperate phage counts may reflect a variety of life history states. Intracellular temperate phage counts could represent prophage DNA still integrated in the host genome, replicating temperate phage that have have excised from the host genome, or temperate phage that entered directly into the lytic cycle immediately post-infection (in eclipse phase). Presuming that the temperate phage signal in the virus-enriched fraction represents packaged phage particles, ~98% of putative temperate phages appeared to have the ability to produce viral particles in situ (Fig. S6b, Table S3). Hence, the lower VC ratios found in the temperate phages does appear to reflect their different lifestyles and reproductive strategies, relative to non-temperate phages.

### Environmental distribution of viral AMGs

Viral copies of AMGs potentially encode for rate-limiting steps in host metabolism that are essential for viral propagation (reviewed in [13]). We show here that the abundance of three key AMGs in energy and nutrient acquisition vary with depth, potentially indicating cellular host variability or energy or nutrient limitation at specific depths.

**Fig. 6:**
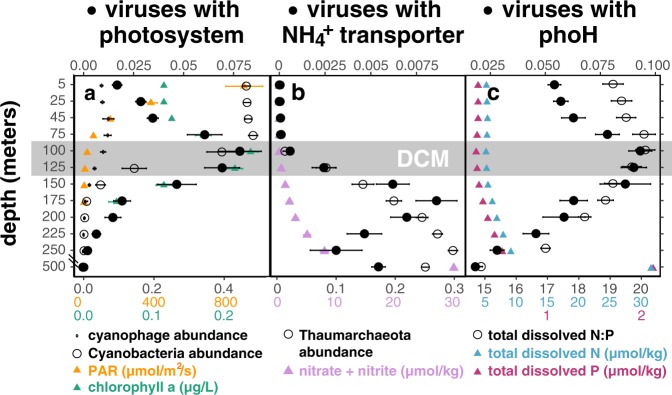
Distribution of viruses containing specific auxiliary metabolic genes (AMGs) in the water column. Solid circles show depth profiles, averaged through time (mean ± SE), of the abundance-normalized proportion of virus populations carrying auxiliary metabolic genes respectively for carbon, nitrogen, and phosphorus metabolism: **a** photosystem, **b** ammonium transporter, and **c** phoH. Time-averaged depth profiles of the following are included for environmental context: **a** relative abundances of cyanophages in the virus-enriched fraction (small open circles) and of cyanobacteria in the cell-enriched fraction (large open circles), photosynthetically active radiation (PAR) (yellow triangles), and fluorometric chlorophyll a (green triangles); **b** Thaumarchaeal relative abundance in the cell-enriched fraction (open circles) and inorganic nitrogen concentrations (purple triangles). c. total dissolved nitrogen (blue triangles), phosphorus (red triangles), and their ratio (open circles). Environmental metadata were retrieved from Hawaii Ocean Time-Series HOT-DOGs application. Gray box highlights depths around the deep chlorophyll maximum (DCM).

Virus-encoded copies of photosystem reaction center genes were first observed in a *Synechococcus* phage genome, were thought to prevent photoinhibition by supplementing declining host photosynthesis during infection [74], and were found to be co-expressed with high-light gene in cyanophages infecting high-light *Prochlorococcus* hosts [4, 75]. Photosystem genes were subsequently observed in many cyanophage genomes [76], yet their environmental distribution in the water column remains relatively unexplored. Here, we observed a threefold increase in abundance of phage carrying photosystem genes from the surface waters to low-light environments around the DCM (Fig. 6a). Considering that cyanophages and cyanobacteria were most abundant in the surface ocean (Figs. 6a, S8a), a higher proportion of cyanophages carried photosystem genes at the DCM relative to surface waters. Our observations suggest that virus-encoded photosystem genes may be more advantageous in light-limited conditions, potentially to prevent light-energy limitation in hosts during infection. Alternatively, longer latent periods, consistent with our observed increase in viral DNA in the cell-enriched fraction at the DCM (Fig. 1a), might also favor phage-encoded photosystem genes at this depth [76].

Viral-encoded ammonium transporter genes, which shared highest similarity to bacterial homologs (Table S9), increased at and below the DCM in tandem with Thaumarchaeota abundance (Fig. 6b). Ammonium-oxidizing Thaumarchaeota in the ocean have demonstrated high affinity for ammonia [77] and therefore may compete with bacteria in ammonia-limited open-ocean environments [78]. As a result, bacteriophage-encoded copies of ammonium transporter genes might assist in ammonium acquisition during infection, particularly at depths with higher abundances of Thaumarchaeota competitors.

PhoH genes are used during phosphorus starvation [79], upregulated during cyanophage infection [80], present in diverse groups of marine viruses [5, 7, 81–83], and can be used as a marker gene for assessing viral assemblages [26, 84]. Viruses are richer in phosphorus relative to cells [85], potentially driving an enrichment of this auxiliary metabolic gene in marine viruses [84] that inhabit relatively nutrient-limited open oceans. At Station ALOHA, up to 10% of viruses encoded copies of phoH genes, peaking at the DCM in tandem with a maximum in the total dissolved nitrogen to phosphorus ratio (Fig. 6c). Our observations suggest that the phoH gene might assist in phosphorus acquisition during viral infection in relatively phosphorus-limited environments.

### Spatiotemporal distributions: temporal persistence and depth distributions

Many virioplankton populations displayed temporal persistence through the 1.5-year time series (Figs. 3, S9), consistent with recent studies at Station ALOHA [26, 32]. However, some virioplankton populations occurred only at specific times of year (Fig. S10), potentially reflecting the seasonal variability of physiochemical variables and their cellular hosts. Of the 9579 viral populations that recruited reads from the ALOHA 2.0 cell-enriched samples from 2014–2016, 6959 (76%) also recruited reads from cell-enriched samples from 2010–2011 (Table S3). This strong temporal persistence indicates that viral genome evolution may be under stabilizing selection, constrained to very specific loci, or occur at finer resolutions than 95% ANI used here for read-mapping and defining populations. Our results show that many indigenous phage populations persist over multiannual cycles, indicative of continuous infection, replication, and lytic cycles that maintain this persistence.

Viral assemblages displayed stratified depth structure, with shifting depth ranges along the transition zone between surface and mesopelagic waters (Figs. 3, S9). This depth structure likely reflects biogeochemical gradients [86] and depth partitioning of bacterial host communities [46]. Consistent with a previous study at Station ALOHA [26], we found no evidence of eurybathic viruses in either the cellular or virioplankton enriched size fractions.

### Depth distributions of archaeal viruses and their hosts

Several different archaeal lineages, including Euryarchaeota and Thaumarchaeota (formerly Group I marine Crenarchaeota [87]) are commonly found along the water column at Station ALOHA [59, 88, 89]. Euryarchaeota apparently function as organic matter degrading heterotrophs, while Thaumarchaeota appear to function as chemolithoautotropic ammonium oxidizers [78, 90]. We identified 161 putative archaeal virus populations, 12 of which represented complete genomes. We classified 67 of these populations as euryarchaeal and 60 as thaumarchaeal, with the latter encompassing crenarchaeal populations (Table S3). In both size fractions, total archaeal virus abundance peaked at and below the DCM (Fig. S8b), with putative thaumarchaeal viruses driving most of this change. Relative abundances of archaeal viruses were consistent, though somewhat lower in magnitude, with that of archaea (Fig. S8b), suggesting that our methods of archaeal virus identification were conservative but accurate.

### Putative *Crocosphaera* phages

The unicellular cyanobacterium *Crocosphaera* can fix both nitrogen and carbon, and plays an important role in fueling primary production in nutrient-poor open ocean environments [91]. Although it is the third-most abundant cyanobacterium at Station ALOHA, after *Prochlorococcus* and *Synechococcus* [52], no *Crocosphaera* phage sequences have yet been identified in current databases. We identified the genome of a putative *Crocosphaera* phage or phage parasite based on multiple lines of evidence: sequence similarity to *Crocosphaera* in 11 of 15 proteins, similar spatiotemporal abundance profiles to that expected from *Crocosphaera* (summer in the upper ocean), and presence during a confirmed *Crocosphaera* “bloom” near Station ALOHA around the same time [52]. This genome contains a higher GC content (41.7%) than other co-occurring viruses (37.3%), consistent with the higher GC content of *Crocosphaera* at ~37.4% [92], compared with abundant surface *Prochlorococcus* at ~32% [46]. Accounting for up to 2% of viral DNA in the virus-enriched size fraction, this genome likely represents a phage, plasmid, or phage-inducible chromosomal island (PICI) packaged into phage particles. PICIs are phage parasitic DNA elements that can hijack another infecting phage’s packaging machinery and mobilize in infectious phage-like particles [93, 94]. The genome (11 kbp) appears complete and encodes a phage integrase (Fig. S6), characteristic of both temperate phages and PICIs. In addition to this genome, we identified six other potential *Crocosphaera* phages (Table S3) based on similar Station ALOHA abundance profiles to *Crocosphaera*, their presence during a *Crocosphaera* “bloom” in one set of samples, and their dissimilarity from *Prochlorococcus* or *Synechococcus* gene homologs.

## Conclusion

Viruses impact the ecology and biogeochemistry of microbial communities across the oceans. Our study recovered a large fraction of dsDNA viruses found at Station ALOHA, revealing a hotspot for microbial diversity at the base of the euphotic zone, where the majority of viruses were distinct from those previously reported. The concurrent characterization of both intracellular and extracellular viruses provided independent support of temperate phage identification using marker genes, and revealed community-wide shifts in viral reproductive strategies. In this open-ocean environment, lytic interactions dominated the upper photic layer above the DCM, and temperate phages were most abundant in the mesopelagic ocean. Temporal variability in reproductive strategies appeared most prevalent in transitional depths at the base of the euphotic zone, marked by a peak in prophage integration and induction. Environmental distributions of viral AMGs also displayed depth-specific patterns in energy and nutrient acquisition. For example, photosystem genes were most abundant at light-limited depths around the DCM; bacteriophage-encoded ammonium transporter genes increased along with potential ammonia-oxidizing thaumarchaeal competitors; phosphorus starvation genes increased in tandem with N:P ratio. Although most viruses were temporally persistent over several years, some displayed temporal variability that appeared to reflect the seasonal distributions of specific hosts. These temporal patterns, in conjunction with other lines of evidence, led to the identification of putative *Crocosphaera* phages or phage parasites that have not been previously identified. Taken together, these new data and analyses provide new insight on the spatiotemporal patterns of planktonic viral diversity, reproductive strategies and metabolic repertoires in the open ocean. Furthermore, simultaneous ennumeration of extracellular and intracellular viruses and their hosts sets the stage for delineating more specific host-virus interactions.

## Supplementary information

Supplementary methods

Supplementary legends

Figure S1

Figure S2

Figure S3

Figure S4

Figure S5

Figure S6

Figure S7

Figure S8

Figure S9

Figure S10

Table S1

Table S2

Table S3

Table S4

Table S5

Table S6

Table S7

Table S8

Table S9

Table S10
