# Supplementary material for: Double-stranded DNA virioplankton dynamics and reproductive strategies in the oligotrophic open ocean water column
Source: ISME J. 2020 Feb 14;14(5):1304–15. doi: 10.1038/s41396-020-0604-8 (PMC7174320; doi:10.1038/s41396-020-0604-8)
Supplement: Supplementary file 1 — Supplementary methods [file 41396_2020_604_MOESM1_ESM.docx]

Double-stranded DNA virioplankton dynamics and reproductive strategies in the oligotrophic open ocean water column

Elaine Luo, John M. Eppley, Anna E. Romano, Daniel R. Mende, Edward F. DeLong

**Supplementary Methods**

Cell-enriched DNA was extracted as previously described (27). Virus-enriched DNA was extracted as previously described (28) with the following modifications. 500μl of sucrose lysis buffer containing 40mM EDTA, 50 mM Tris pH 8.3, 0.75M sucrose, and 0.5 mg/mL lysozyme were added to the filter housing for a 30 minute incubation at 37°C. Then, 200μl of sucrose lysis buffer containing 0.8 mg/mL of Proteinase K and 1% SDS were added for a 2 hour incubation at 55°C. Lysates were recovered and stored at -80°C for up to a week prior to batch purification. Frozen lysates were thawed at room temperature, incubated at 55°C for 10 minutes, then DNA was purified on an automated magnetic bead purification instrument MSM using Chemagen DNA Saliva buffer kit (Perkin Elmer CMG-1037-1, Waltham MA).

DNA was sheared using AFA-micro-15 (Covaris 520145, Woburn MA) following Illumina’s Neoprep protocol. Fragment sizes were quantified using the Fragment Analyzer (Agilent DNF-488-0500 Santa Clara CA) to customize Covaris M220 shear times to 350bp. Libraries were prepared using Neoprep droplet liquid handler and TruSeq Nano LT kits (Illumina NP-101-1001, San Diego CA) for 350bp insert size. Libraries were quantified using Picogreen (Thermo Fisher P11496, Waltham MA) and normalized to equal concentrations prior to pooling. For virus-enriched libraries, 24 samples were multiplexed for each NextSeq 500 sequence run (Illumina FC- 404-2004, San Diego CA) targeting 17 million paired-end reads per sample at 150bp. Cell-enriched libraries were sequenced as previously described (29) targeting 33 million paired-end reads per sample at 150bp. 1% PhiX was added for quality control.

Reads were demultiplexed using Illumina’s Bcl2fastq v2.17.1.14 and quality filtered in two steps using the bbduk.sh script from bbmap v36.84 (30). The first step removed sequencing adapters with options “ktrim=r k=23 mink=11 hdist=1 tbo tpe tbo tpe ref=resources/adapters.fa”, and the second step removed phiX and low quality sequences with options “k=27 hdist=1 qtrim=rl trimq=17 cardinality=t mingc=0.05 maxgc=0.95 ref=resources/phix174_ill.ref.fa.gz”. Reads were then filtered with bfc vr181, with options "-1 -k 21," to correct or remove unique kmers (31).
