# Supplementary material for: Double-stranded DNA virioplankton dynamics and reproductive strategies in the oligotrophic open ocean water column
Source: ISME J. 2020 Feb 14;14(5):1304–15. doi: 10.1038/s41396-020-0604-8 (PMC7174320; doi:10.1038/s41396-020-0604-8)
Supplement: Supplementary file 2 — Supplementary legends [file 41396_2020_604_MOESM2_ESM.docx]

Double-stranded DNA virioplankton dynamics and reproductive strategies in the oligotrophic open ocean water column

Elaine Luo, John M. Eppley, Anna E. Romano, Daniel R. Mende, Edward F. DeLong

**Supplementary legends**

**Figure S1.** **Bioinformatic workflow.** Data products included in this publication are highlighted in black.

**Figure S2.** **Hydrography depth profile time-series of samples collected during the Hawaii Ocean Time-Series and used to prepare the ALOHA2.0 virus database.** Points represent time and depth of 186 samples taken for the 0.2-0.02μm virus-enriched size fraction. Lines in panels a and f respectively represent the mixed layer depth (surface density offset of 0.125) and euphotic zone depth (1% of surface PAR). Data obtained via the Hawaii Ocean Time-Series HOT-DOGS application, University of Hawai'i at Mānoa, National Science Foundation Award #1756517 (http://hahana.soest.hawaii.edu/hot/hot-dogs).

**Figure S3.** **Cut-off selection for identifying putative archaeal viruses.** The proportion of proteins without RefSeq84 hits (y-axis) and the ratio of top hits to Archaea/archaeal virus vs. Bacteria (AB ratio, x-axis) was calculated for a. all possible archaeal viruses with one or proteins with top hit to to Archaea or archaeal virus on RefSeq, and b. high-confidence archaeal viruses containing archaeal protein markers from PFAM (bit score >30). Based on the high-confidence archaeal viruses in b, we used cutoffs of >0.8 proportion of unknown proteins and >0.5 AB ratio to refine populations in a., retaining 147 putative archaeal viruses shown in c. Combining b. and c. resulted in a final set of 161 putative archaeal viruses.

**Figure S4.** Examples of viral genomes in the ALOHA 2.0 database. Top captions represent contig number, putative hosts based on the highest number of protein hits to reference genome, genome completion, and size. The outer ring represents ALOHA 2.0 viruses, and the inner ring represents reference genomes with 10 or more protein hits. Blue shading between the genomes represent amino acid identity from LAST alignments. Genes of interest are color-coded by functional groups (PFAM bit score >30).

**Figure S5. Genomic structure of a putative *Crocosphaera* phage or phage parasite.** Top caption represent contig number, putative host, genome completion, and size. Genes of interest are color-coded by functional groups (PFAM bit score >30) described in Fig. S5. Taxonomic and functional annotations are included for RefSeq84, PFAM, and EggNOG databases.

**Figure S6.** **Size fraction distribution and genome sizes of putative temperate phage and other viruses.** a. Median VC ratio across all samples, relative abundance profiles in the b. virus-enriched and c. cell-enriched size fractions, and d. genome sizes of 1543 complete circular viral populations. Inferred temperate phages are shown in blue, while all other populations are shown in orange.

**Figure S7.** **Depth profiles of time-averaged proportion (mean +/-SE) of putative temperate SAR11 phages normalized to all SAR11 phages.** Open circles represent phages captured in the cell-enriched fraction, and closed circles represent phages captured in the virus-enriched fraction.

**Figure S8. Virus-host relative abundances for a. cyanophage and b. thaumarchaeal virus.** Time-averaged depth profiles (mean +/-SE) show viruses in the virus-enriched size fraction (small closed circles), viruses in the cell-enriched size fraction (large closed circles), and hosts in the cell-enriched size fraction (open circles).

**Figure S9. Spatiotemporal distributions of all virus populations present in the virus-enriched size fraction.** Each node on the top dendrogram and its associated column represents the coverage profile of one virus population. The green bar near the dendrogram represents 171 populations displaying summer blooms in the upper ocean, representing possible *Crocosphaera* phages for downstream identification. Rows represent individual samples that are horizontally ordered by depth and time. The height of the black bar in every sample shows mean coverage (calculated using only the second and third quartile) of every population, normalized to the maximum coverage in that sample.

**Figure S10. Temporal variability in relative abundances of select annotated virus populations at 5m, 125m, and 250m, and their correlation with environmental variables:** potential temperature (temp), fluorometric chlorophyll a (chl), Prochlorococcus+Synechecoccus abundance (cyano), heterotrophic bacterial abundance (hbact), nitrate+nitrite (nit), and phosphate (phos). Stars represent significant Spearman’s rho correlations (P<0.05). White shading indicates missing data.

**Table S1.** Sample information and associated metadata. Sample naming conventions are as follows: HSD[size fraction]-[HOT cruise number]-[depth in meters]-[cast number]-[sequencing date in yymmdd]. Data obtained via the Hawaii Ocean Time-Series HOT-DOGS application, University of Hawai'i at Māno, National Science Foundation Award #1756517 (http://hahana.soest.hawaii.edu/hot/hot-dogs).

**Table S2.** Sequencing, initial assemblies, VIRSorter contigs, and read statistics for all samples. Columns in order represents sample name, number of quality-controlled reads in each sample, number of contigs assembled by metaSPAdes, number of contigs >3kb, number of >3kb contigs that was identified as viral (all categories) by VIRSorter, and number of viral reads mapping to VIRSorter-identified contigs. Two smaller columns on the right represent the number of viral reads summed across depth. Sampling points with multiple sequencing runs were included at this initial stage prior to viral-specific reassembly. In post-reassembly analyses, only the largest sequencing run for any sampling point was used. Sample naming conventions are described in Table S1.

**Table S3.** Information for 17 369 >10kbp ALOHA 2.0 virus populations: name, GC content, length, circularity, name of circular genome representative (if redundant), chimeric signature, temperate phage identification, homology (>60% AAI across >50% genes) to RefSeq84 viruses or viral metagenomic datasets (names consistent with Fig. 1), modified homology (any AAI across >50% genes) to RefSeq84 viruses, archaeal virus identification, eukaryotic virus identification, *Crocosphaera* phage identification, presence in virus-enriched fraction, presence in cell-enriched fraction, and presence in 2010-1 dataset.

**Table S4.** Relative abundances of 17 369 ALOHA 2.0 virus populations in the virus-enriched fraction, approximated by nucleotides mapping to population normalized to nucleotides mapping to all populations. Sample naming conventions are described in Table S1.

**Table S5.** Relative abundances of 17 369 ALOHA 2.0 virus populations in the cell-enriched fraction, approximated by nucleotides mapping to population normalized to nucleotides mapping to all populations. Sample naming conventions are described in Table S1.

**Table S6.** Relative abundances of 1543 circular ALOHA 2.0 virus populations in the virus-enriched fraction, approximated by the population’s coverage normalized to summed coverage across all populations. Sample naming conventions are described in Table S1.

**Table S7.** Relative abundances of 1543 circular ALOHA 2.0 virus populations in the cell-enriched fraction, approximated by the population’s coverage normalized to summed coverage across all populations. Sample naming conventions are described in Table S1.

**Table S8.** Relative abundances of 2568 mOTUS (cellular populations) in the cell-enriched fractions, approximated by the population’s coverage normalized to summed coverage across all populations. Sample naming conventions are described in Table S1, with the exception of omitted sequencing date.

**Table S9.** Taxonomic assignments (top hits) of ALOHA 2.0 virus proteins aligned using LAST to the RefSeq84 protein database.

**Table S10.** List of novel viral PFAM domains (bit score >30) from ALOHA 2.0 populations that are distinct from two reported lists in previous metagenomic datasets (13,25).
