# Supplementary figures and images for: Double-stranded DNA virioplankton dynamics and reproductive strategies in the oligotrophic open ocean water column
Source: ISME J. 2020 Feb 14;14(5):1304–15. doi: 10.1038/s41396-020-0604-8 (PMC7174320; doi:10.1038/s41396-020-0604-8)

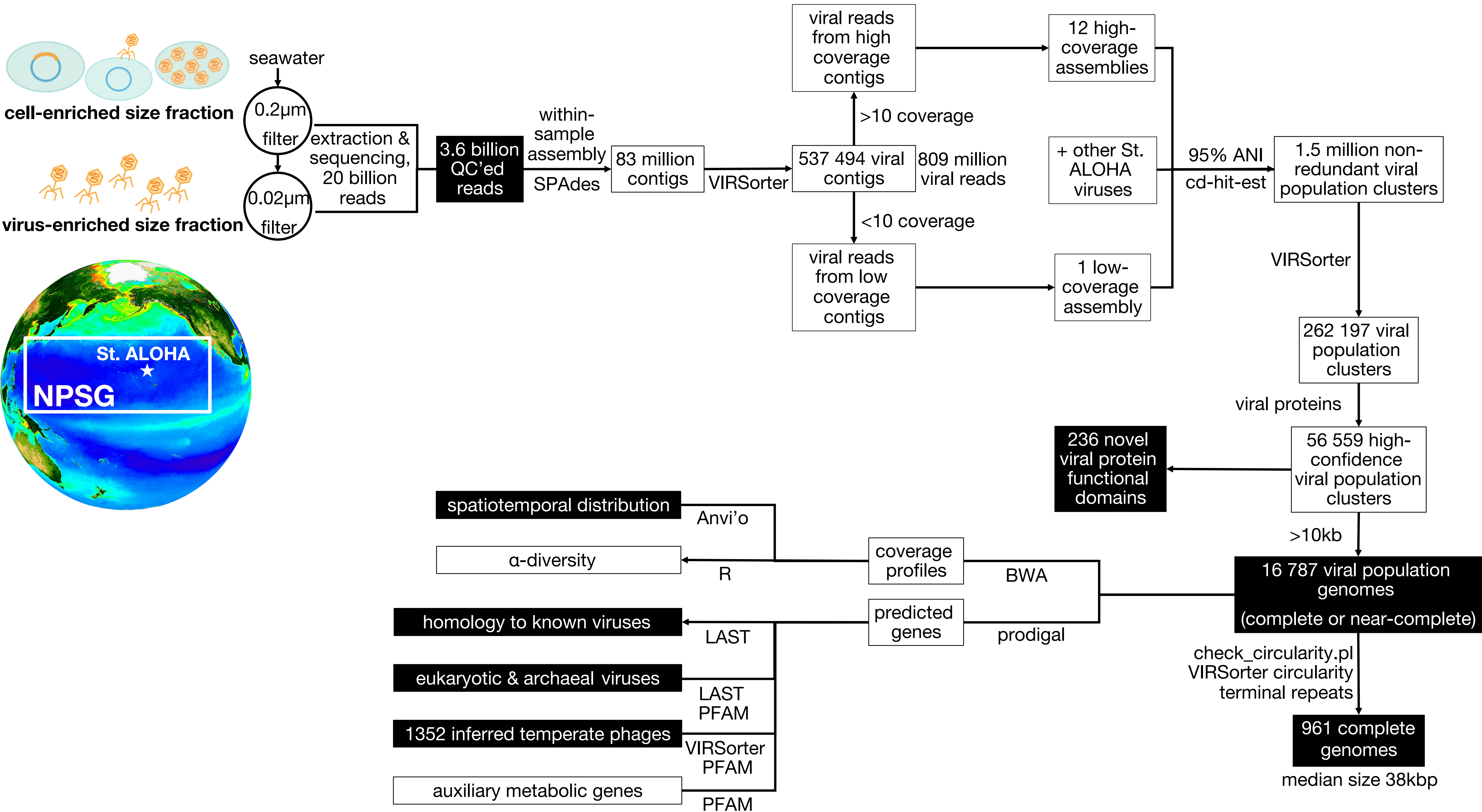

Supplement: Supplementary file 3 — Figure S1 [file 41396_2020_604_MOESM3_ESM.pdf]

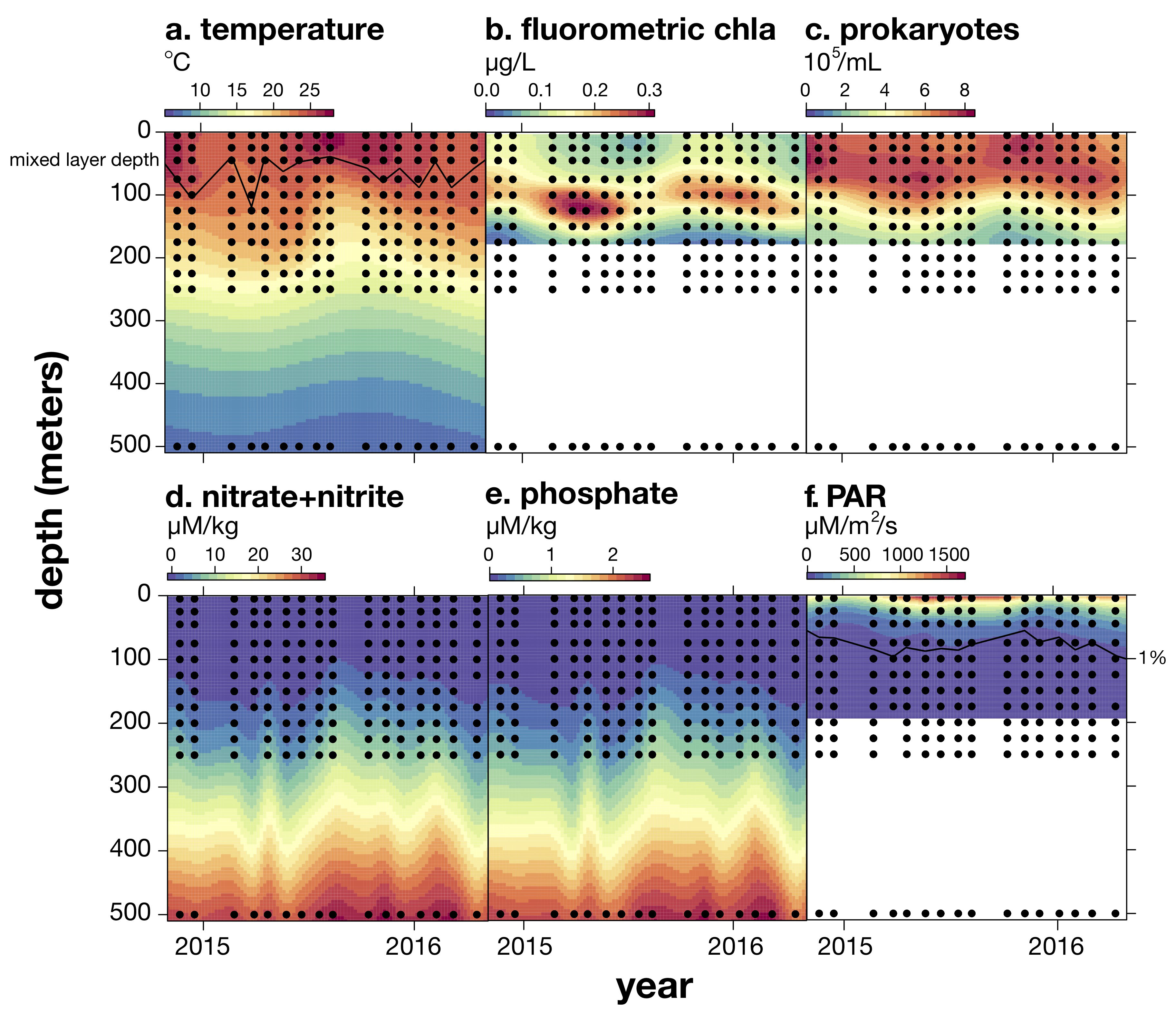

Supplement: Supplementary file 4 — Figure S2 [file 41396_2020_604_MOESM4_ESM.jpg]

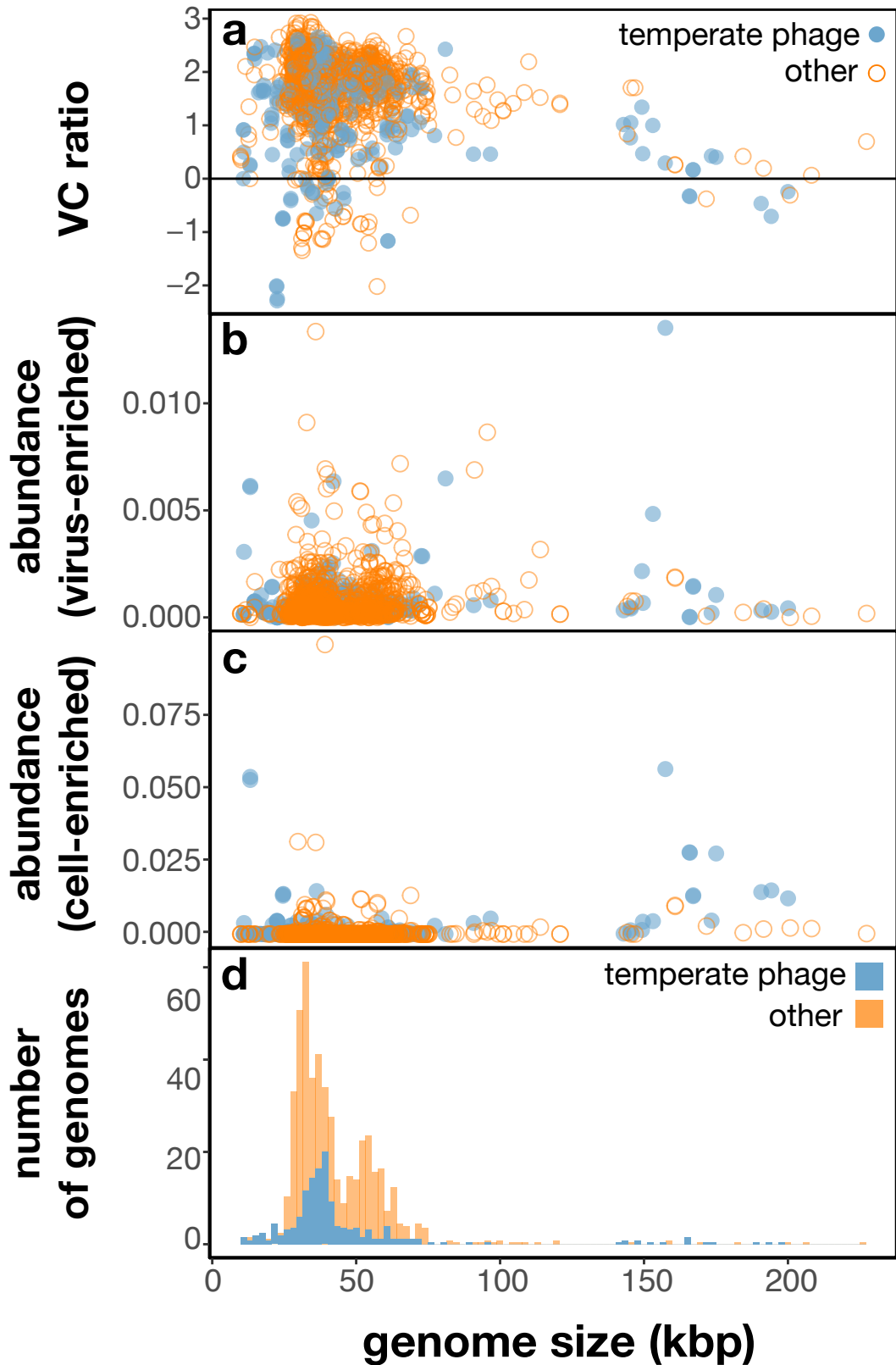

Supplement: Supplementary file 8 — Figure S6 [file 41396_2020_604_MOESM8_ESM.pdf]

# SAR11 phage

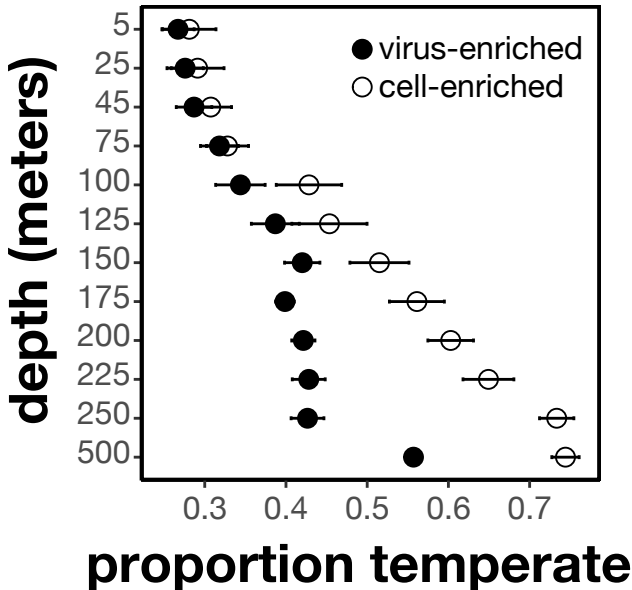

Supplement: Supplementary file 9 — Figure S7 [file 41396_2020_604_MOESM9_ESM.pdf]

### a. cyanophage

### b. thaumarchaeal virus

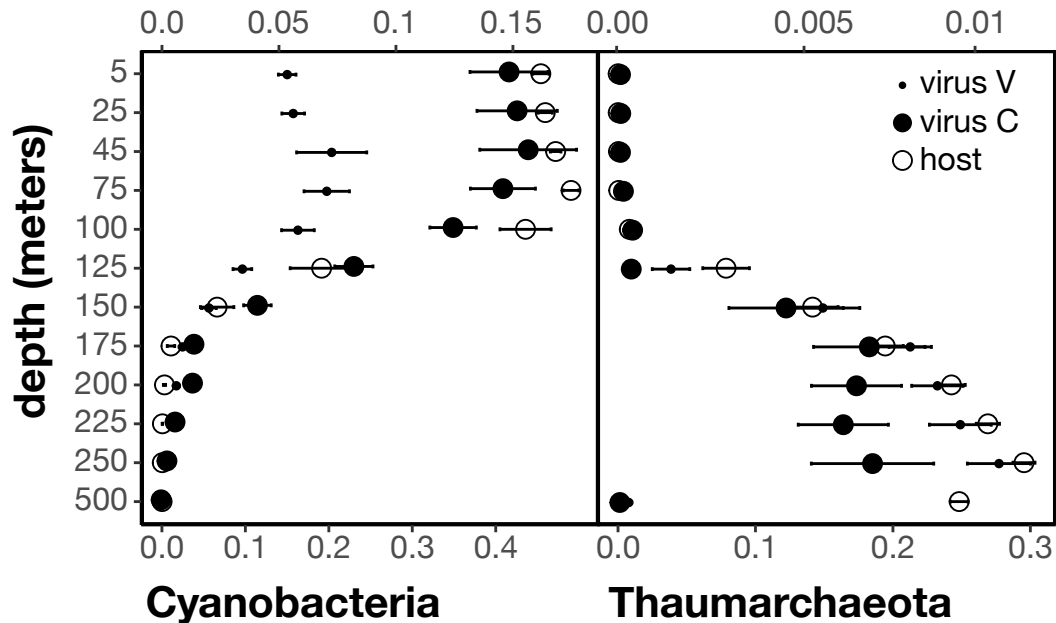

Supplement: Supplementary file 10 — Figure S8 [file 41396_2020_604_MOESM10_ESM.pdf]

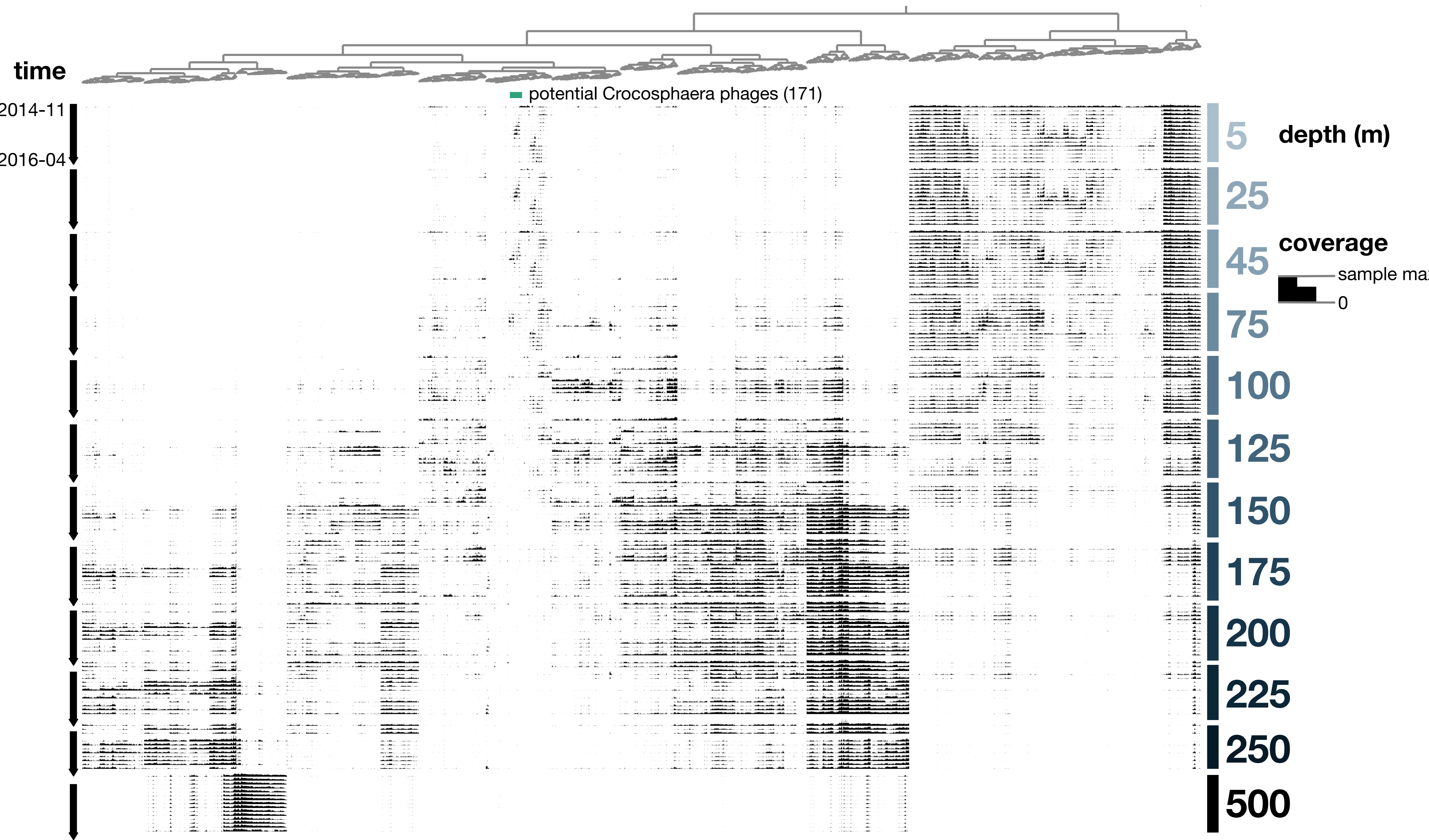

Supplement: Supplementary file 11 — Figure S9 [file 41396_2020_604_MOESM11_ESM.pdf]
