# Supplementary material for: Double-stranded DNA virioplankton dynamics and reproductive strategies in the oligotrophic open ocean water column
Source: ISME J. 2020 Feb 14;14(5):1304–15. doi: 10.1038/s41396-020-0604-8 (PMC7174320; doi:10.1038/s41396-020-0604-8)
Supplement: Supplementary file 5 — Figure S3 [file 41396_2020_604_MOESM5_ESM.pdf]

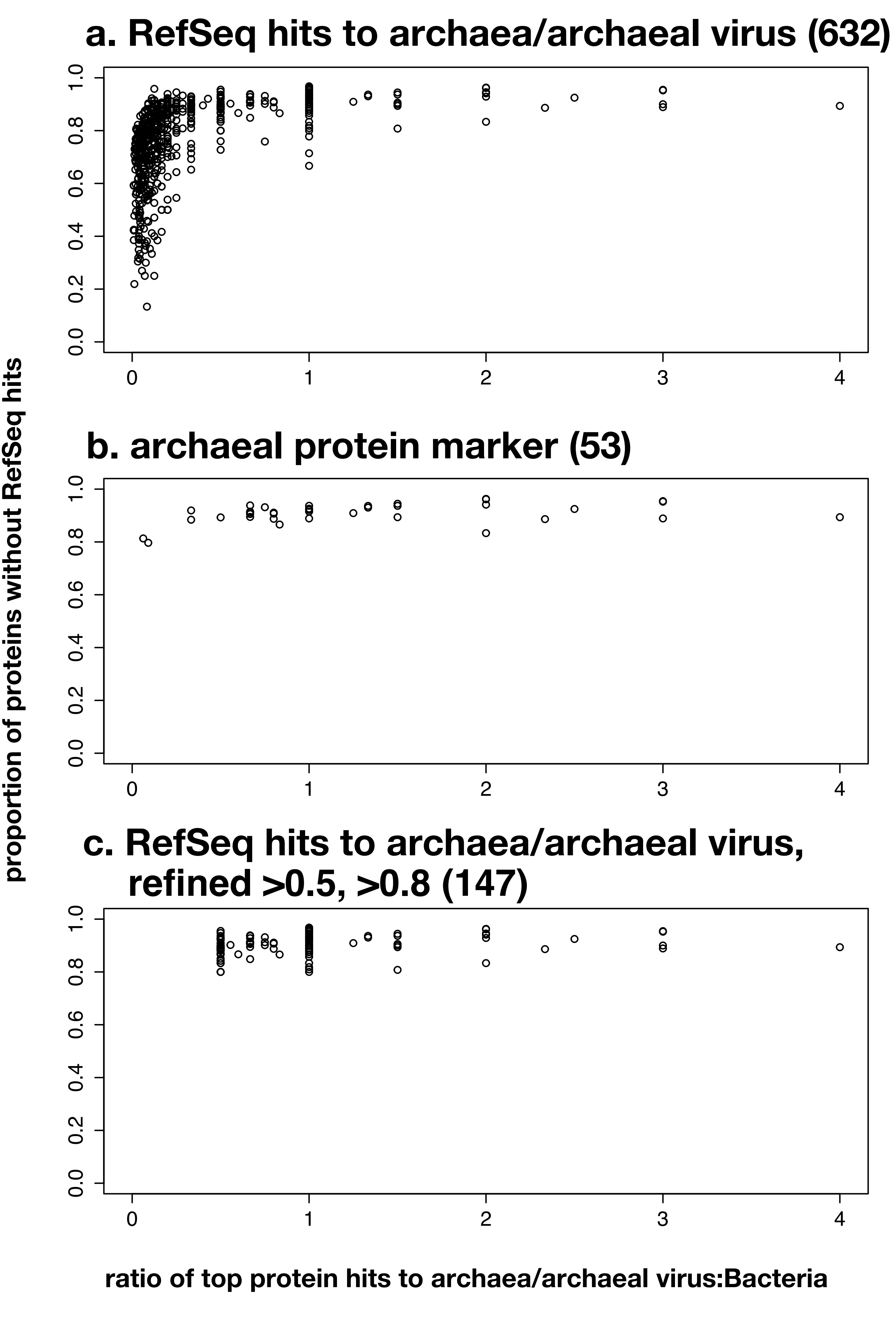

a. RefSeq hits to archaea/archaeal virus (632)
b. archaeal protein marker (53)
c. RefSeq hits to archaea/archaeal virus, refined >0.5, >0.8 (147)
proportion of proteins without RefSeq hits
ratio of top protein hits to archaea/archaeal virus:Bacteria
0.0
0.2
0.4
0.6
0.8
1.0
0
1
2
3
4
