# Supplementary material for: Double-stranded DNA virioplankton dynamics and reproductive strategies in the oligotrophic open ocean water column
Source: ISME J. 2020 Feb 14;14(5):1304–15. doi: 10.1038/s41396-020-0604-8 (PMC7174320; doi:10.1038/s41396-020-0604-8)
Supplement: Supplementary file 6 — Figure S4 [file 41396_2020_604_MOESM6_ESM.pdf]

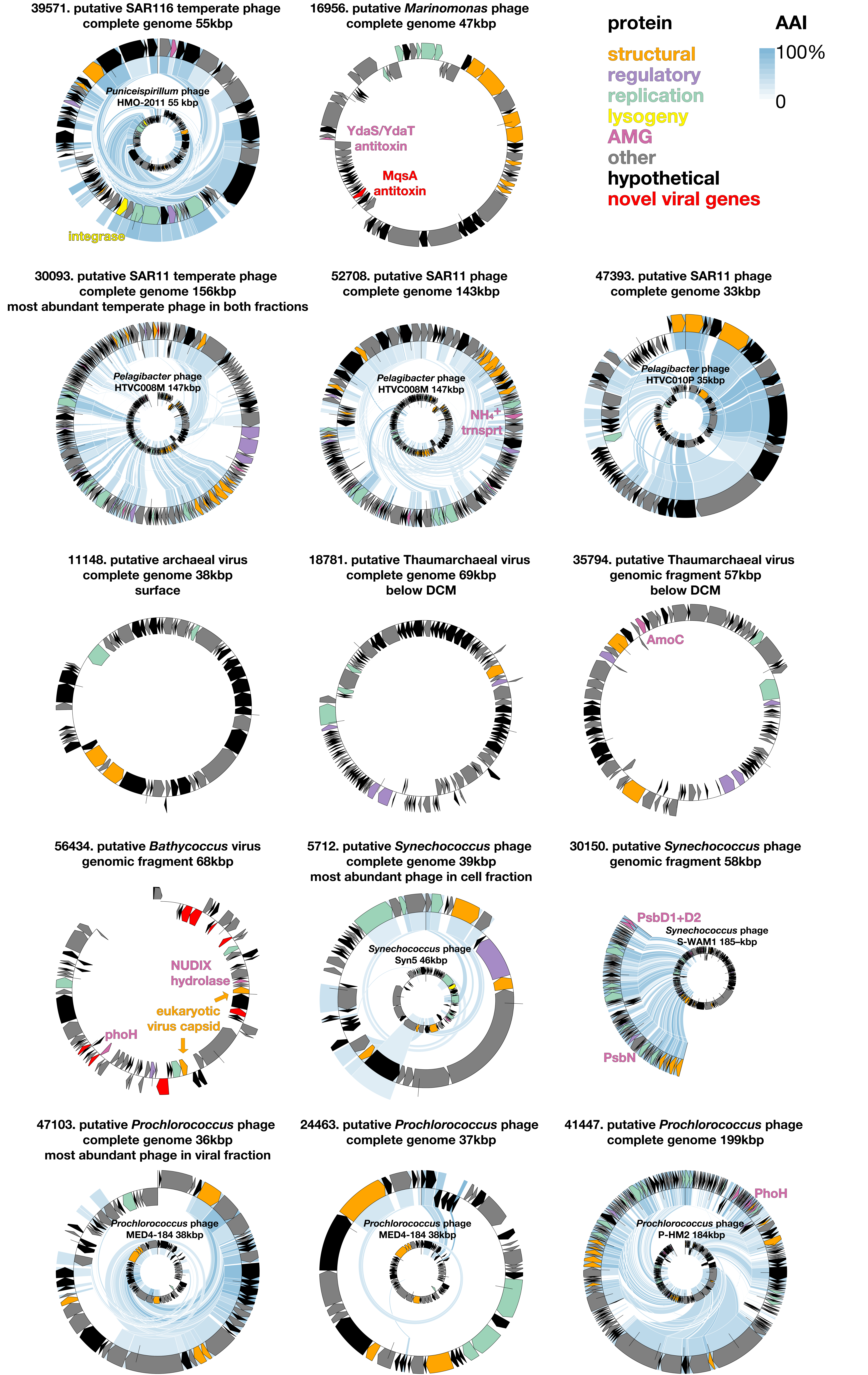

39571. putative SAR116 temperate phage
complete genome 55kbp
Puniceispirillum phage
HMO-2011 55 kbp
integrase
16956. putative Marinomonas phage
complete genome 47kbp
YdaS/YdaT
antitoxin
MqsA
antitoxin
protein
structural
regulatory
replication
lysogeny
AMG
other
hypothetical
novel viral genes
AAI
100%
0
30093. putative SAR11 temperate phage
complete genome 156kbp
most abundant temperate phage in both fractions
Pelagibacter phage
HTVC008M 147kbp
52708. putative SAR11 phage
complete genome 143kbp
Pelagibacter phage
HTVC008M 147kbp
NH4+
trnsprt
47393. putative SAR11 phage
complete genome 33kbp
Pelagibacter phage
HTVC010P 35kbp
11148. putative archaeal virus
complete genome 38kbp
surface
18781. putative Thaumarchaeal virus
complete genome 69kbp
below DCM
35794. putative Thaumarchaeal virus
genomic fragment 57kbp
below DCM
AmoC
56434. putative Bathycoccus virus
genomic fragment 68kbp
NUDIX
hydrolase
eukaryotic
virus capsid
phoH
5712. putative Synechococcus phage
complete genome 39kbp
most abundant phage in cell fraction
Synechococcus phage
Syn5 46kbp
30150. putative Synechococcus phage
genomic fragment 58kbp
PsbD1+D2
Synechococcus phage
S-WAM1 185-kbp
PsbN
47103. putative Prochlorococcus phage
complete genome 36kbp
most abundant phage in viral fraction
Prochlorococcus phage
MED4-184 38kbp
24463. putative Prochlorococcus phage
complete genome 37kbp
Prochlorococcus phage
MED4-184 38kbp
41447. putative Prochlorococcus phage
complete genome 199kbp
PhoH
Prochlorococcus phage
P-HM2 184kbp
