# Supplementary material for: Double-stranded DNA virioplankton dynamics and reproductive strategies in the oligotrophic open ocean water column
Source: ISME J. 2020 Feb 14;14(5):1304–15. doi: 10.1038/s41396-020-0604-8 (PMC7174320; doi:10.1038/s41396-020-0604-8)

11140. putative *Crocosphaera* phage, complete genome 11kbp

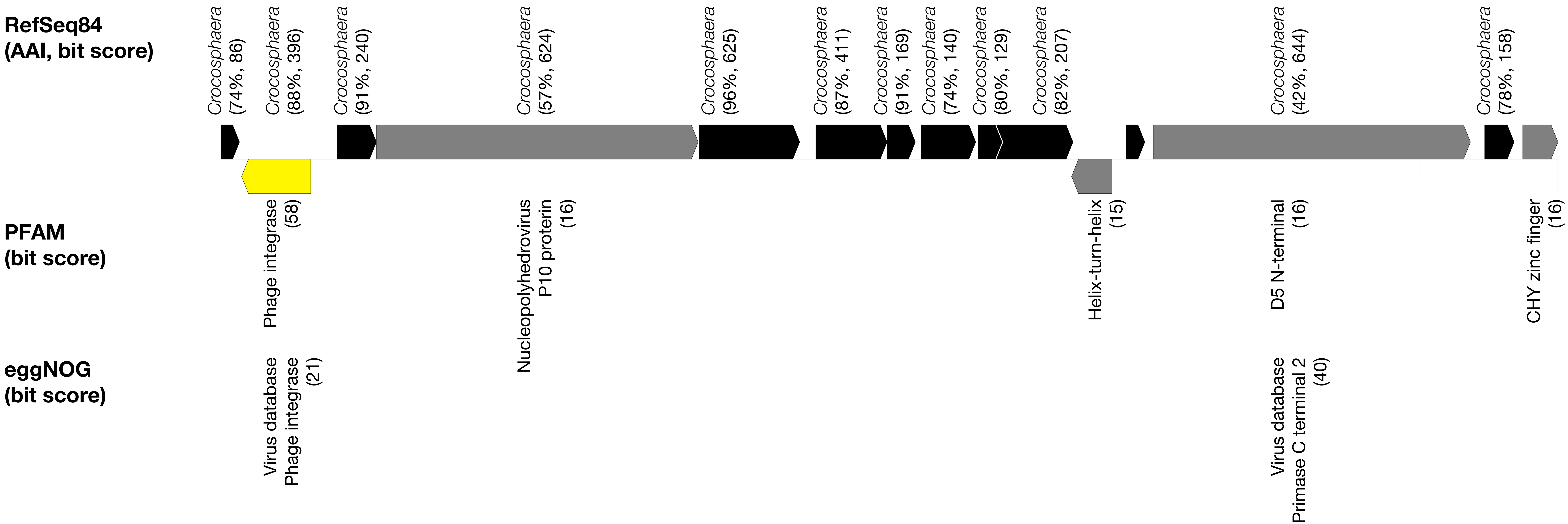

Supplement: Supplementary file 7 — Figure S5 [file 41396_2020_604_MOESM7_ESM.pdf]
