# Supplementary material for: Double-stranded DNA virioplankton dynamics and reproductive strategies in the oligotrophic open ocean water column
Source: ISME J. 2020 Feb 14;14(5):1304–15. doi: 10.1038/s41396-020-0604-8 (PMC7174320; doi:10.1038/s41396-020-0604-8)
Supplement: Supplementary file 12 — Figure S10 [file 41396_2020_604_MOESM12_ESM.pdf]

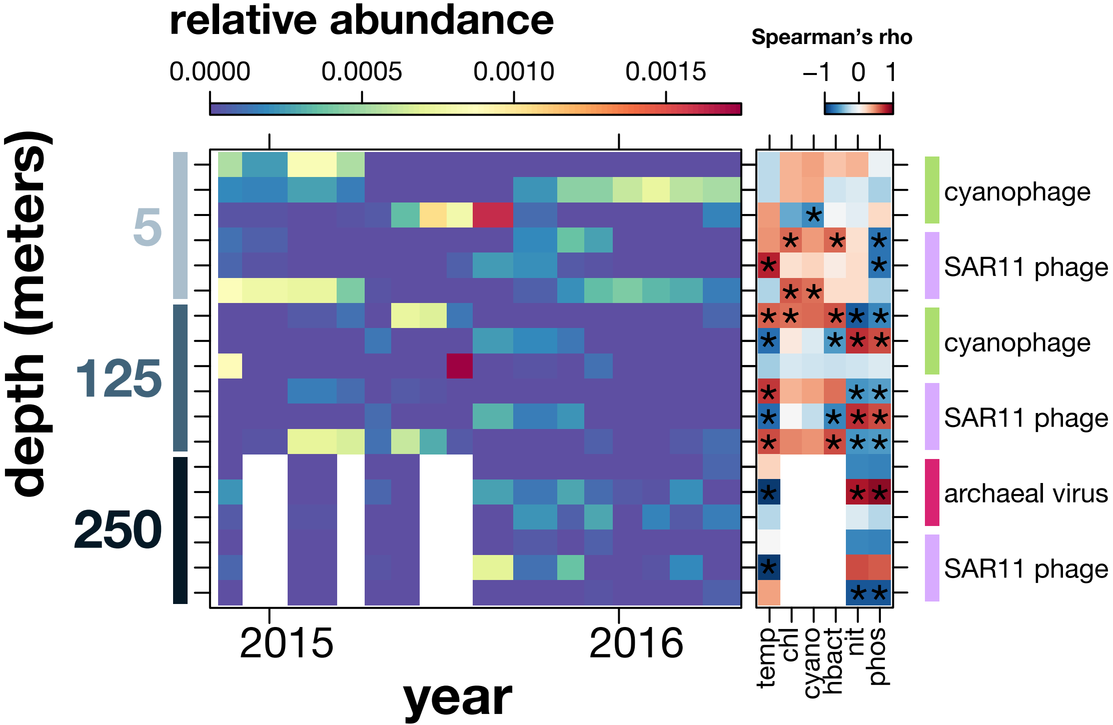
relative abundance
0.0000
0.0005
0.0010
0.0015
Spearman's rho
−1
0
1
depth (meters)
5
125
250
2015
2016
year
temp
chl
cyano
hbact
nit
phos
cyanophage
SAR11 phage
cyanophage
SAR11 phage
archaeal virus
SAR11 phage
